# Early sleep and sensory over-responsivity relate to insular functional connectivity with sensorimotor cortices and cerebellum in infants at varying likelihood for autism

**DOI:** 10.1186/s11689-026-09721-0

**Published:** 2026-08-04

**Authors:** Emily Chiem, Lauren Wagner, Leanna M. Hernandez, Shulamite Green, Mirella Dapretto

**Affiliations:** 1 Molecular, Cellular, and Integrative Physiology Program, University of California, Los Angeles, Los Angeles, CA 90095 USA; 2 Neuroscience Interdepartmental Program, University of California, Los Angeles, Los Angeles, CA 90095 USA; 3 Department of Psychiatry and Biobehavioral Sciences, University of California, Los Angeles, Los Angeles, CA 90095 USA; 4 Center for Autism Research and Treatment, University of California, Los Angeles, Los Angeles, CA 90095 USA; 5 Department of Psychology, University of California, Los Angeles, Los Angeles, CA 90095 USA; 6 Ahmanson-Lovelace Brain Mapping Center, University of California, Los Angeles, Los Angeles, CA 90095 USA

**Keywords:** Infant, Sleep problems, Sensory processing, fMRI, Autism spectrum disorder, Insula

## Abstract

Sleep problems and sensory over-responsivity (SOR) are common, co-occurring, and early emerging features of Autism Spectrum Disorder (ASD). Yet, the early neural mechanisms underlying these symptoms remain unclear. Here, we used resting-state fMRI data from the Infant Brain Imaging Study (IBIS) to examine how functional brain connectivity at 6 months may relate to parent-reported measures of sleep-onset problems and SOR in infants at varying familial likelihood for ASD. The right anterior insula (rAI) – a key hub of the salience network – was used in ROI-ROI analyses to investigate functional connectivity to cortical and cerebellar regions previously implicated in sleep disruption, sensory processing challenges, and ASD. Infants at high (HL) and low (LL) likelihood for ASD displayed divergent patterns of rAI connectivity with sensorimotor cortex, as well as cerebellar regions involved in sensorimotor processing and higher-order functions. Furthermore, stronger rAI connectivity with sensorimotor cortices and cerebellar regions was associated with worse sleep-onset problems and SOR in HL infants. In contrast, stronger rAI-cerebellar connectivity was related to fewer sleep-onset problems and SOR in LL infants. Our findings indicate that altered rAI connectivity may result in over-attribution of attention to sensory stimuli and highlight aberrant sensory prediction learning, which may underlie worse sleep problems and higher SOR in HL infants.

**Supplementary Information:**

The online version contains supplementary material available at https://doi.org/10.1186/s11689-026-09721-0.

## Introduction

Sleep problems are pervasive in Autism Spectrum Disorder (ASD), occurring in up to 80% of children with ASD [1] and are some of the first concerns raised by parents prior to a formal diagnosis [2]. While the core diagnostic criteria for ASD include social-communication difficulties and restrictive, repetitive behaviors [3], sleep disruption is another common feature that persists throughout the lifespan with profound consequences on cognition and overall daily functioning [4, 5]. Prior work has shown that sleep disruption in infancy has negative effects on developmental outcomes, such as cognition [6–8], language [9, 10], motor skills [11], and even later ASD traits [12, 13]. Yet, our understanding of the impact of sleep problems on neurodevelopment, particularly in the first few years of life, remains limited. To our knowledge, only one study has investigated how infant sleep disturbances relate to brain development in ASD [14]. This study showed that difficulties with sleep initiation in the first year of life are associated with slower growth trajectories in subcortical brain volume from 6 to 24 months in infants who later received an ASD diagnosis. Despite the high prevalence of sleep problems in autism and their potential to impact later developmental outcomes, the relationship between sleep problems in infancy and functional brain connectivity in ASD has yet to be examined.

Sensory processing challenges were added as a core symptom of ASD in the DSM-V [3] and can emerge as early as infancy, prior to a formal diagnosis [15]. These sensory atypicalities often manifest as sensory over-responsivity (SOR) – characterized by extreme responses to or avoidance of sensory stimuli [16, 17]. As these sensory challenges can cause extreme discomfort, they have been identified as a research priority by those in the autistic community [18]. Past work has also shown that toddlers with ASD display increased SOR compared to toddlers without a diagnosis [19–21] and that these sensory atypicalities are negatively associated with later adaptive behavior [22]. Notably, several studies have demonstrated a significant co-occurring, positive association between sleep problems and sensory processing differences in children and adolescents with ASD [23–28], such that worse sleep problems are related to increased sensory responsivity. Furthermore, both sleep problems and sensory challenges are related to higher levels of ASD traits, such as social functioning [29, 30] and repetitive behaviors [31, 32]. The correlation between autism-associated sleep problems and sensory atypicalities consistently reported from toddlerhood to adolescence suggests that these symptoms may be linked throughout development.

Although the co-occurrence of sleep and sensory processing challenges in ASD has been well-documented, the early neural mechanisms underlying these associations have yet to be elucidated. Toddlers are typically among the youngest age groups investigated in sleep and sensory studies of ASD [16, 25], despite the fact that the biological bases for sleep and sensory problems are likely disrupted during the fetal and/or neonatal period. While several theories have been proposed to explain the relationship between sleep disturbances and sensory responsivity in ASD, the underlying mechanisms are likely complex and multifactorial. Some have suggested that poor sleep may exacerbate core ASD symptoms, such as difficulty regulating sensory inputs [33, 34]. On the other hand, because the sensory gating process largely modulates sleep [35], the inability to properly attenuate sensory inputs may disrupt sleep onset and sleep maintenance [36]. Given the intertwined and reciprocal nature of sleep and sensory processes, early perturbations in their potentially shared neural mechanisms may exacerbate these symptoms and contribute to altered neurodevelopment.

The brain’s functional connectome emerges *in utero* [37]. Large-scale functional connectivity networks undergo dramatic development during infancy [38, 39], with the salience network (SN) – a functional network involved in detecting and prioritizing relevant stimuli [40] – detectable in the first few months of life [41]. SN connectivity is consistently altered in individuals with ASD. For instance, hyperconnectivity between core SN nodes, including the right anterior insula (rAI), has been observed in children with ASD and was related to more severe restricted and repetitive behaviors [42]. Other work in adults with ASD has implicated both hypo- and hyperconnectivity of the SN, including decreased connectivity between the prefrontal cortex and cerebellum, and increased connectivity between temporoparietal regions [43]. Even earlier in development, recent work has shown that stronger connectivity between the salience and visual networks was associated with higher levels of social impairment in school-aged children at varying likelihood for ASD [44]. In addition, stronger SN connectivity with sensorimotor cortices is related to worse sensory over-responsivity in youth with ASD [45, 46], suggesting that there may be an over-attribution of attention to extraneous sensory stimuli.

While three studies to date have investigated SN connectivity in infants at increased familial likelihood for ASD [47–49], the links between SN connectivity, sleep problems, and sensory challenges in this infant population have yet to be investigated. Since 20% of infants who have an older sibling with ASD ultimately receive a diagnosis [50], prospective studies in this population provide a unique opportunity to examine how early functional brain atypicalities may predict later ASD traits and diagnostic outcomes. Indeed, several studies have shown early structural [51, 52] and functional [53–56] brain differences in these infant siblings, which predicted later ASD traits [57–62]. Furthermore, parents of infants at high familial likelihood for ASD report greater sleep problems, such as longer sleep latency and decreased nighttime sleep duration [63]. Recent work has shown that 6-week-old infants at high likelihood for ASD exhibit stronger SN connectivity to sensorimotor regions – which was predictive of later sensory sensitivities – and weaker connectivity to prefrontal regions in comparison to infants with no familial predisposition for ASD [47]. This altered SN connectivity pattern may reflect impairments in detecting and orienting towards salient stimuli, and supports the hypothesis that individuals with ASD may perceive low-level sensory inputs as more salient than socially relevant information [64]. Together, this suggests that differences in salience detection may manifest early in development.

In addition to changes in SN connectivity, ASD has also been associated with altered cerebellar functional connectivity [65–70]. Previous work has shown that children and adolescents with ASD show greater cerebellar functional connectivity with cortical sensorimotor networks [65] and that connectivity between these regions is associated with SOR symptoms [70]. In the context of a sparse, but growing, literature on the role of the cerebellum in infant behavioral development [71], very few studies (e.g [72–74]), have focused on functional cerebellar connectivity, particularly in ASD. Functional cerebellar networks are present soon after birth [75], and cerebro-cerebellar networks have been defined in neonates [74] and 6-month-old infants [76]. Although studies of cerebellar functional connectivity in infants at familial likelihood for ASD are sparse, one prior study in 9-month-olds [72] has investigated the right cerebellar crus I, which is implicated in language processing and working memory [77, 78]. They show that that weaker connectivity between the right cerebellar crus I and frontal cortex was associated with delayed language trajectories in infants at varying likelihood for ASD [72]. In addition, emerging evidence shows that the cerebellum is involved in sleep [79], particularly in sleep-dependent memory consolidation [80]. The cerebellum, an important structure in error-based and predictive learning [81], continues to process motor information during sleep. Thus, it has been posited that the sleeping cerebellum continues to update its internal models [82], and suggests that sleep problems may disrupt the refinement of cerebellar predictive learning. Overall, these findings position the cerebellum at the intersection between sleep, sensory processing, and ASD.

Despite independent evidence linking SN and cerebellar functional connectivity to ASD and sleep disturbances [80, 82–85], it remains unclear whether alterations in these networks are associated with both sleep difficulties and sensory processing atypicalities in early life. Here, we used functional magnetic resonance imaging (fMRI) to examine the early neural correlates of sleep problems and SOR in infants at high (HL; older sibling with a diagnosis) and low (LL; no family history) likelihood for ASD. We examined how functional connectivity between the right anterior insula (rAI) – a main hub of the SN – with cortical and cerebellar regions of interest at 6 months of age may relate to emerging sleep-onset problems and SOR symptoms. Based on prior literature showing atypical brain connectivity in HL infants and ASD youth, our a priori regions of interest included sensorimotor cortices [45, 47] and regions of the cerebellum involved in sensorimotor processing (lobules I-IV, lobules V-VI, lobules VIIIA/B; [70]) and higher-order functions (crus I; [72]). In addition, we examined how sleep-onset problems and SOR symptoms may be associated with later ASD-related traits.

## Methods

### Participants

Participants were enrolled as part of the Infant Brain Imaging Study (IBIS). Publicly available data was downloaded from the National Institute of Mental Health Data Archive (Collection #19 and #2027). The IBIS is a longitudinal study aimed at investigating early brain and behavioral development in infants at varying familial likelihood for ASD. High likelihood (HL) infants had at least one older sibling with a confirmed ASD diagnosis. Low likelihood (LL) infants had at least one older typically developing sibling and no first- or second-degree relatives with ASD or an intellectual disability. Parents provided written informed consent, and infants were screened and assessed at each data collection site: University of North Carolina at Chapel Hill, University of Washington, Children’s Hospital of Philadelphia, or Washington University in St Louis. Research protocols were approved by the institutional review boards at each site. As defined by the IBIS protocols, participants were excluded based on the following criteria: premature birth (gestational age < 36 weeks) or low birth weight (< 2,000 g); significant medical or neurological conditions affecting growth, development, or cognition; maternal substance use during pregnancy; a first degree relative with psychosis, schizophrenia, or bipolar disorder; contraindication for MRI; non-English-speaking family; adoption; and twin birth [60].

A total of 101 infants (62 HL, 39 LL) with fMRI scans and Infant Behavior Questionnaire (IBQ) data, both collected at 6 months of age, were included in the present study. Demographics are summarized in Table 1. Groups were matched on sex, age at scan, race, ethnicity, and maternal education. Due to attrition common in longitudinal studies, only seventy-nine infants also had IBQ data collected at 12 months. Data from the Sensory Experiences Questionnaire (SEQ) was available at 12 months (*N* = 76) and at 24 months (*N* = 63). Eighty-eight infants received a clinical best-estimate diagnosis of ASD at 24 or 36 months based on the Diagnostic and Statistical Manual of Mental Disorders, fourth edition, text revision (DSM-IV-TR) criteria using all available testing and interview data including the Autism Diagnostic Observation Schedule (ADOS; [86]), the Autism Diagnostic Interview-Revised (ADI-R; [87]), and the Mullen Scales of Early Learning [88]. In the present HL sample, only 7 infants received an ASD diagnosis at 24 months of age. Due to the small sample size of infants diagnosed with ASD, all HL infants were treated as a uniform group.

**Table 1:** Sample demographics and behavioral scores

	HL (mean ± SD)	LL (mean ± SD)	t or χ2
*N*	62	39	-
Sex (# female)	22	15	0.01 (*p* = 0.93)
Scan Age (months)	5.89 ± 0.65	5.97 ± 0.48	0.77 (*p* = 0.45)
Race ^a^			
* White*	49	32	0.99 (*p* = 0.32)
* Non-white or mixed-race*	11	3	
Ethnicity ^a^			
* Hispanic*	3	2	< 0.001 (*p* = 1) ^b^
* Non-Hispanic*	57	33	
Maternal Education ^a^			
* College degree & above*	42	31	3.30 (*p* = 0.07)
* No college degree*	18	4	
Scanner head motion			
* Mean relative motion (mm)*	0.068 ± 0.036	0.07 ± 0.03	0.30 (*p* = 0.77)
* Mean absolute motion (mm)*	0.162 ± 0.139	0.210 ± 0.183	1.40 (*p* = 0.17)
* # ICA components removed*	13.2 ± 4.71	12.7 ± 3.59	0.67 (*p* = 0.51)
ISOP Score			
* 6 months (N)*	2.72 ± 1.22 (62)	2.48 ± 0.793 (39)	1.20 (*p* = 0.23)
* 12 months (N)*	2.67 ± 1.04 (55)	2.47 ± 0.937 (24)	0.86 (*p* = 0.40)
SOR Score			
* 12 months (N)*	17.1 ± 5.01 (54)	17 ± 2.67 (22)	0.19 (*p* = 0.85)
* 24 months (N)*	17.4 ± 4.70 (44)	18.7 ± 2.31 (19)	1.48 (*p* = 0.26)
ADOS (24–36 months)	2.41 ± 2.31	1.25 ± 0.508	3.61 (*p* < 0.001) *
MSEL T-Scores			
* ELC*	93.8 ± 17.5	110 ± 15.5	4.80 (*p* < 0.001) *
* Gross Motor*	46 ± 12.4	51.7 ± 11.7	2.37 (*p* = 0.02) *
* Fine Motor*	48.1 ± 9.56	55.4 ± 9.65	3.73 (*p* < 0.001) *
* Visual Reception*	49.8 ± 11.2	55.3 ± 10.6	2.47 (*p* = 0.02) *
* Receptive Language*	45.1 ± 14	55.5 ± 8.58	4.65 (*p* < 0.001) *
* Expressive Language*	43.4 ± 11.4	53.3 ± 10.6	4.45 (*p* < 0.001) *

Higher Infant Sleep-Onset Problems (ISOP) scores indicate worse problems. Higher Sensory Over-Responsivity (SOR) scores indicate higher severity SOR behavior

* Signifies statistical significance

^a^ Unknown or unreported race, ethnicity, and maternal education information for 2 HL and 3 LL infants

^b^ Fishers Exact Test used to confirm statistical non-significance

*ICA *Independent Component Analysis, *ADOS *Autism Diagnostic Observation Schedule, *MSEL *Mullen Scales of Early Learning, *ELC *Early Learning Composite

### Behavioral measures and assessments

For the present study, we used several measures related to cognitive development, adaptive functioning, and behaviors associated with ASD which were among the battery of questionnaires and assessments collected as part of the IBIS. The Infant Behavior Questionnaire Revised (IBQ-R), collected at 6 and 12 months of age, assessed temperament along 14 scales [89]. Since the original IBIS dataset did not include a sleep-specific questionnaire or assessment, five questions related to sleep-onset and settling down from the IBQ-R were summed to generate the Infant Sleep-Onset Problems (ISOP) score (Supplementary Table S1), as performed and validated in a prior study [14]. The Sensory Experiences Questionnaire (SEQ), a parent-reported measure of a child’s sensory processing patterns, was collected at 12 and 24 months [90]. A sensory over-responsivity (SOR) score was generated based on summed raw scores for auditory, visual, and tactile items that related to SOR symptoms (Supplementary Table S2). While the SEQ has a hyper-responsiveness scale, it also includes scores from one Gustatory/Olfactory question, one Vestibular/Proprioceptive question, and one addendum item on “picky” eating. Given that auditory [23], visual [91], and tactile [24] domains have reported associations with sleep problems in ASD, we restricted our SOR score to these domains. Additional analyses were conducted with the broader SEQ hyper-responsiveness scale to facilitate comparisons with prior studies. The Mullen Scales of Early Learning, collected at 6, 12, and 24 months, assessed language, motor, cognitive and perceptual abilities [88]. For most infants, age-normed scores from the 24-month assessment (*N* = 85) were used. For infants without 24-month scores, scores from 6-month (*N* = 7) or 12-month (*N* = 9) assessments were used. The Autism Diagnostic Observation Schedule (ADOS), collected at 24 or 36 months was used to evaluate ASD symptoms [86].

### MRI data acquisition

MRI scans were collected during natural sleep at 6 months of age on cross-site calibrated 3T Siemens Tim Trio scanners with 12-channel head coils. In this study, we analyzed 3D T2 TSE (TR = 3,200 ms, TE = 497 ms, 160 sagittal slices, FOV = 256 mm, voxel size = 1 mm^3^) and resting-state scans (5.4 min each) acquired using a gradient echo planar sequence (TR = 2,500 ms, TE = 27 ms, FOV = 256 mm, voxel size = 4 mm^3^, flip angle = 90). One to four resting-state scans were collected for each participant. On average, two scans per participant were retained for analysis following quality control (see *fMRI Data Preprocessing* below) (Supplementary Fig. S1). The number of scans retained for each participant was not weighted in the subsequent analyses.

### fMRI data preprocessing

Functional MRI data were preprocessed and analyzed using FMRIB’s Software Library (FSL version 6.00; [92]). Structural T2 images were skull stripped using FSL’s Brain Extraction Tool, followed by manual inspection. Functional scans underwent motion correction and linear registration to the subject’s corresponding structural image, followed by registration to a standard 6-month infant brain template [93]. Spatial smoothing was performed using a 6-mm Gaussian kernel and then scans underwent 4D mean intensity normalization. Mean relative and absolute motion (mm) did not significantly differ between the two groups (Table 1). Independent Component Analysis – Automatic Removal of Motion Artifacts (ICA-AROMA; [94, 95]) was used to automatically remove components identified as noise or motion, as this has been shown to improve reproducibility of resting-state analyses [96]. High-quality fMRI scans were defined as having mean relative motion < 0.40 mm and > 10 ICA components retained following ICA-AROMA in order to capture the primary resting-state networks in the brain [97]. HL and LL groups did not significantly differ on the number of ICA components removed (Table 1). As ICA-AROMA denoising uses linear regression to remove components identified as noise, all acquired fMRI volumes ([128–129]) were retained for analysis. Furthermore, data were bandpass filtered (0.01 Hz < t < 0.1 Hz) to reduce physiological noise. Mean white matter, cerebrospinal fluid, and global time series were regressed from the data at the single-subject level [98].

### Statistical analysis and fMRI data analysis

Behavioral analyses were performed using R v4.2.1. Group differences in ADOS severity scores and Mullen ELC scores were examined using a Welch two-sample t-test. A Levene’s test was used to assess the significance of variance between the two groups. Spearman rank order correlations were performed to examine the relationship between ISOP (6 and 12 months), SOR (12 and 24 months), and ADOS severity scores (24–36 months). Spearman rank order correlations were selected because they are less sensitive to outliers, relying on relative ranks of the data rather than raw values [99]. Given our a priori hypotheses about the relationships between ISOP, SOR, and ADOS scores, correction for multiple comparisons was not applied. To ensure that relationships were not driven by the cognitive, language, and motor domains captured by Mullen ELC scores, Mullen ELC scores were included as a covariate in follow-up linear regression models with the lme4 package.

**Fig. 1:**
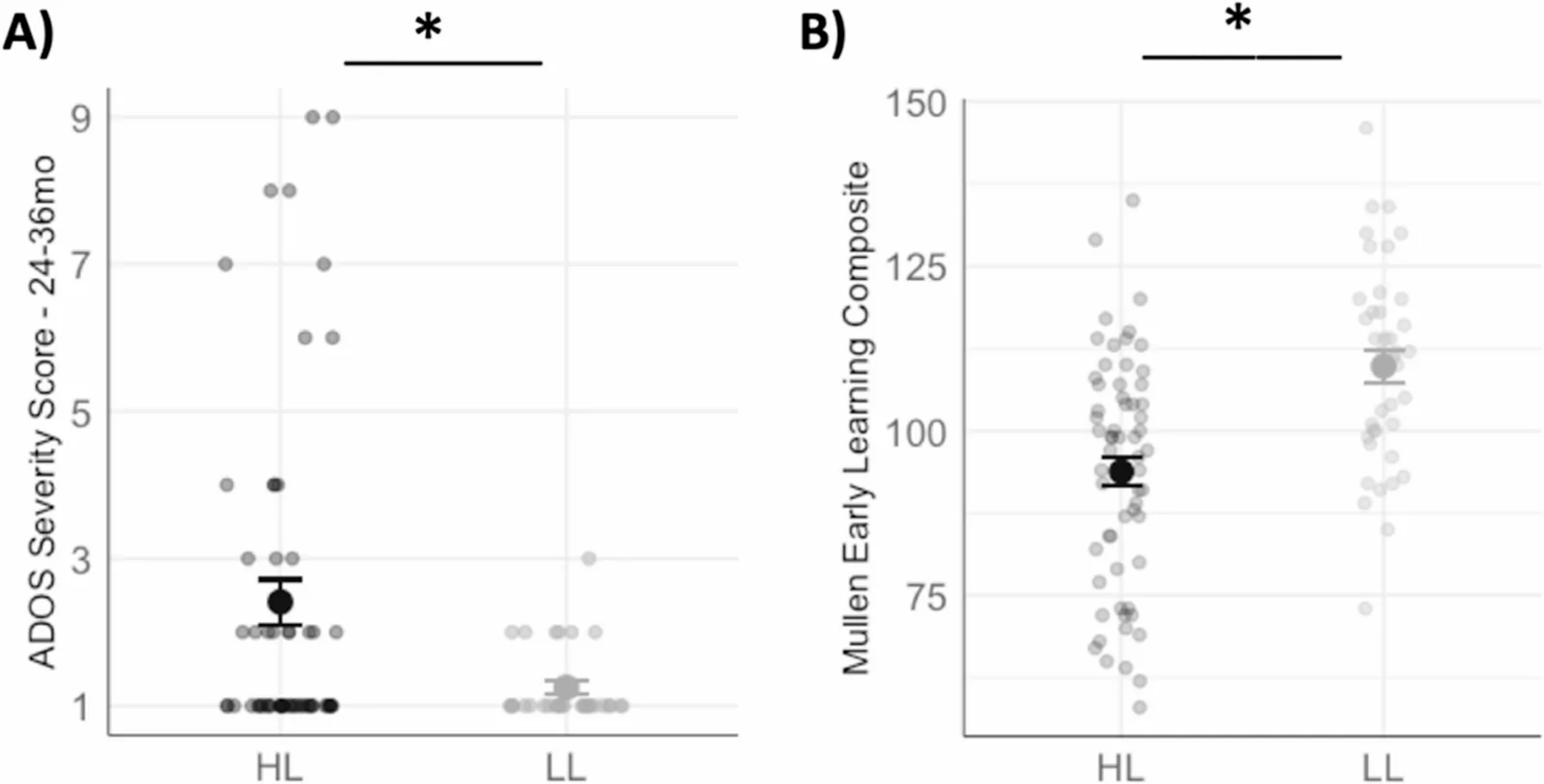
Differences in developmental scores between high likelihood (HL) and low likelihood (LL) infants.** A **Significant group difference in Autism Diagnostic Observation Schedule (ADOS) severity scores from 24–36 months (*p* < 0.001). **B **Significant group difference in Mullen early language composite scores (*p* < 0.001)

All connectivity analyses were anchored at the right anterior insula (rAI) to interrogate SN connectivity, as done in several prior studies [40, 45, 47, 100], including a recent study in a similar population of infants at varying familial likelihood for ASD [47]. The rAI was chosen based on prior work that has shown that the rAI serves as the primary site for inputs to the SN [101] and plays a critical role in activating and deactivating networks in response to salient stimuli [102]. In addition, a meta-analysis has identified consistent differences in rAI activation in individuals with ASD as compared to neurotypical controls [103], suggesting that the rAI is particularly vulnerable in ASD. A rAI mask was derived from an anatomical parcellation of the right insula available from a standard infant atlas [93]. We created a rAI anatomical mask composed of voxels anterior to the midline of the center of gravity of the right insula, as done in a prior study [47]. The time series, averaged across the rAI, was extracted from processed residuals in standard space and correlated with that of every other voxel in the brain to generate single-subject functional connectivity maps. Using Fisher’s r-to-z transformation, the resulting correlation maps were converted to z-statistic maps. Group-level analyses were performed in FSL using FMRIB’s Local Analysis of Mixed Effects (FLAME 1 + 2), within the general linear model (GLM) framework. This models the dependent variable at each voxel as a linear combination of predictors specified in a design matrix. Independent variables of interest included group (HL vs. LL) for between-group contrasts, as well as continuous behavioral measures (ISOP or SOR) for bottom-up behavioral regressions. For significant associations, we conducted additional control analyses with ISOP and SOR included in the same model to identify distinct patterns of association. Specifically, sleep analyses were repeated controlling for SOR, and SOR analyses were repeated controlling for ISOP. Although prior work in this dataset found that data quality did not significantly differ by site [104] and identical scanners were used at each site, we included site as a demeaned nuisance covariate in all models to further minimize any potential site-related variability. Since HL and LL infants differed on the Mullen Early Learning Composite (ELC) scores (Fig. 1B), these were also included as demeaned nuisance covariates in all models. Statistical maps were thresholded at Z > 2.3 (*P* < 0.01) and further corrected using a cluster-defining threshold of *P* < 0.05 to account for multiple voxelwise comparisons, as implemented in FSL. All regression analyses were restricted to anatomically defined regions of interest (ROI), thus limiting the number of voxelwise comparisons. Given our focus on a priori regions previously implicated in sleep disruption and sensory challenges, or shown to be altered in HL infants in prior work [45, 47, 70, 72], we did not perform additional multiple comparison correction across the anatomical ROIs. Our ROIs included cortical areas involved in sensorimotor processing (left and right sensorimotor cortex), cerebellar regions canonically known for their role in sensorimotor processing (bilateral lobules I-IV, bilateral lobules V-VI, and bilateral lobules VIIIA/B; [105–107], and cerebellar regions involved in higher order functions (left and right crus I [77, 78], (Supplementary Fig. S2). Lobules V-VI were distinguished from lobules I-IV due to reports of their distinct functional connectivity with visual and auditory cortices [105, 108], as well as from lobules VIIIA and VIIIB due to their anatomical distance [70]. rAI connectivity the left and right crus I was examined separately, given prior evidence implicating the right crus I in language processing and working memory [77, 78]. Sensorimotor parcellations were derived from the Shi et al. (2011) infant atlas [109]. Cerebellum parcellations were derived from an anatomical map of the adult cerebellum [110]. All parcellations were transformed to standard space before conducting single-subject analyses [93].

## Results

### Behavioral measures

First, we examined whether HL and LL infants differed on infant sleep-onset problems (ISOP), sensory over-responsivity (SOR), and other measures of early behavioral development. There were no significant group differences in ISOP scores at 6 or 12 months (Table 1). However, HL infants showed significantly greater variance in ISOP scores relative to LL infants at 6 months (Levene’s test, *p* = 0.027). There were no significant group differences in SOR scores at 12 or 24 months (Table 1), nor in variance. Groups significantly differed on ASD traits as assessed by the ADOS at 24–36 months (t = 3.61, *p* < 0.001); HL infants had higher scores, and thus greater severity of ASD-related behaviors, compared to LL infants (Fig. 1A). Also, HL infants showed significantly greater variance in ADOS severity scores (Levene’s test, *p* < 0.001) as compared to LL infants, reflecting the heterogeneity of the HL group. The groups differed on the Mullen ELC scores, as well as across the other Mullen subscales (Table 1), such that LL infants had higher scores than HL infants (all t > 2.47, *p* < 0.02) (Fig. 1B). Given the significant group difference in Mullen ELC scores, these were added as a regressor in all subsequent analyses to ensure that any significant findings were not related to differences in early cognitive, language, or motor skills, as indexed by the Mullen ELC.

**Fig. 2:**
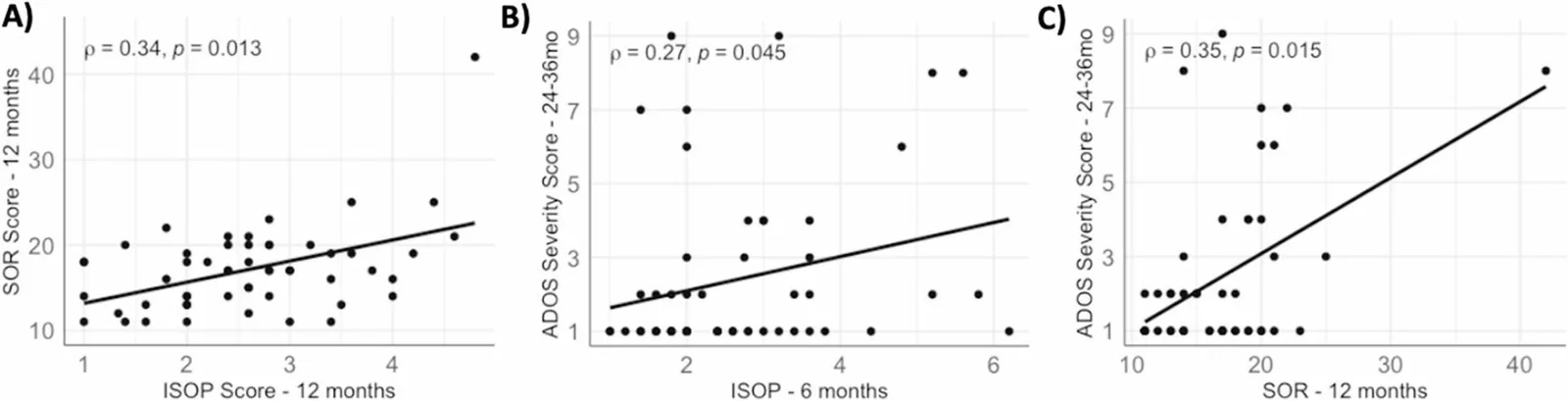
Spearman rank order correlations between Infant Sleep-Onset Problems (ISOP), Sensory Over-responsivity (SOR), and Autism Diagnostic Observation Schedule (ADOS) Scores in high likelihood (HL) infants.** A **Significant correlation between ISOP and SOR at 12 months (*p* = 0.013). **B **Significant correlation between 6-month ISOP and ADOS severity scores at 24–36 months (*p* = 0.045). **C **Significant correlation between 12-month SOR and ADOS severity scores at 24–36 months (*p* = 0.015)

Next, we investigated relationships between sleep-onset problems and sensory over-responsivity, as well as their associations with later ASD traits. Given our specific hypotheses concerning HL infants and the limited range of scores in the LL infants, correlation analyses were restricted to the HL group. At 12 months, HL infants showed a significant relationship between ISOP and SOR scores, such that greater sleep-onset problems were related to higher sensory over-responsivity (Spearman’s *ρ* = 0.34, *p* = 0.013; Fig. 2A). This relationship did not hold with the broader hyper-responsivity scale (Supplementary Fig. S3), suggesting that sleep-onset problems are specifically related to SOR. Additionally, greater SOR scores at 12 months were associated with greater SOR scores at 24 months (Spearman’s *ρ* = 0.38, *p* = 0.011; Table 2). There was no significant association between 6- or 12-month ISOP scores and later SOR scores (Table 2). Finally, we asked how ISOP and SOR related to later ASD traits. In HL infants, greater ISOP scores at 6 months was associated with greater ADOS severity scores at 24–36-month outcome (Spearman’s *ρ* = 0.27, *p* = 0.045; Fig. 2B). Furthermore, HL infants with greater SOR scores at 12 months showed greater ADOS severity scores at 24–36-month outcome (Spearman’s *ρ* = 0.35, *p* = 0.015; Fig. 2C); this relationship held even when the outlier was removed (Spearman’s *ρ* = 0.3, *p* = 0.035), as well as with the broader hyper-responsivity scale (Supplementary Fig. S3), suggesting that overall sensory hyper-responsivity is associated with greater ASD traits. The associations between SOR and ADOS severity scores remained significant and the relationship between ISOP and ADOS severity scores was trending after conducting follow-up regression analyses controlling for Mullen ELC scores (Supplementary Table S3).

**Table 2:** Means, standard deviations, and spearman rank order correlations between ISOP, SOR, and ADOS in high likelihood (HL) infants

Variable	M	SD	1	2	3	4	5
1. ISOP (6mo)	2.72	1.22	-				
2. ISOP (12mo)	2.67	1.04	0.21	-			
3. SOR (12mo)	17.1	5.01	0.02	0.34 *	-		
4. SOR (24mo)	17.4	2.31	0.20	0.11	0.38 *	-	
5. ADOS	2.41	2.31	0.27 *	0.17	0.35 *	0.29	-

Infant Sleep-Onset Problems (*ISOP*); Sensory Over-Responsivity (*SOR*); Autism Diagnostic Observation Schedule (*ADOS*). * *p* < 0.05

### Group differences in right anterior insula (rAI) connectivity

**Fig. 3:**
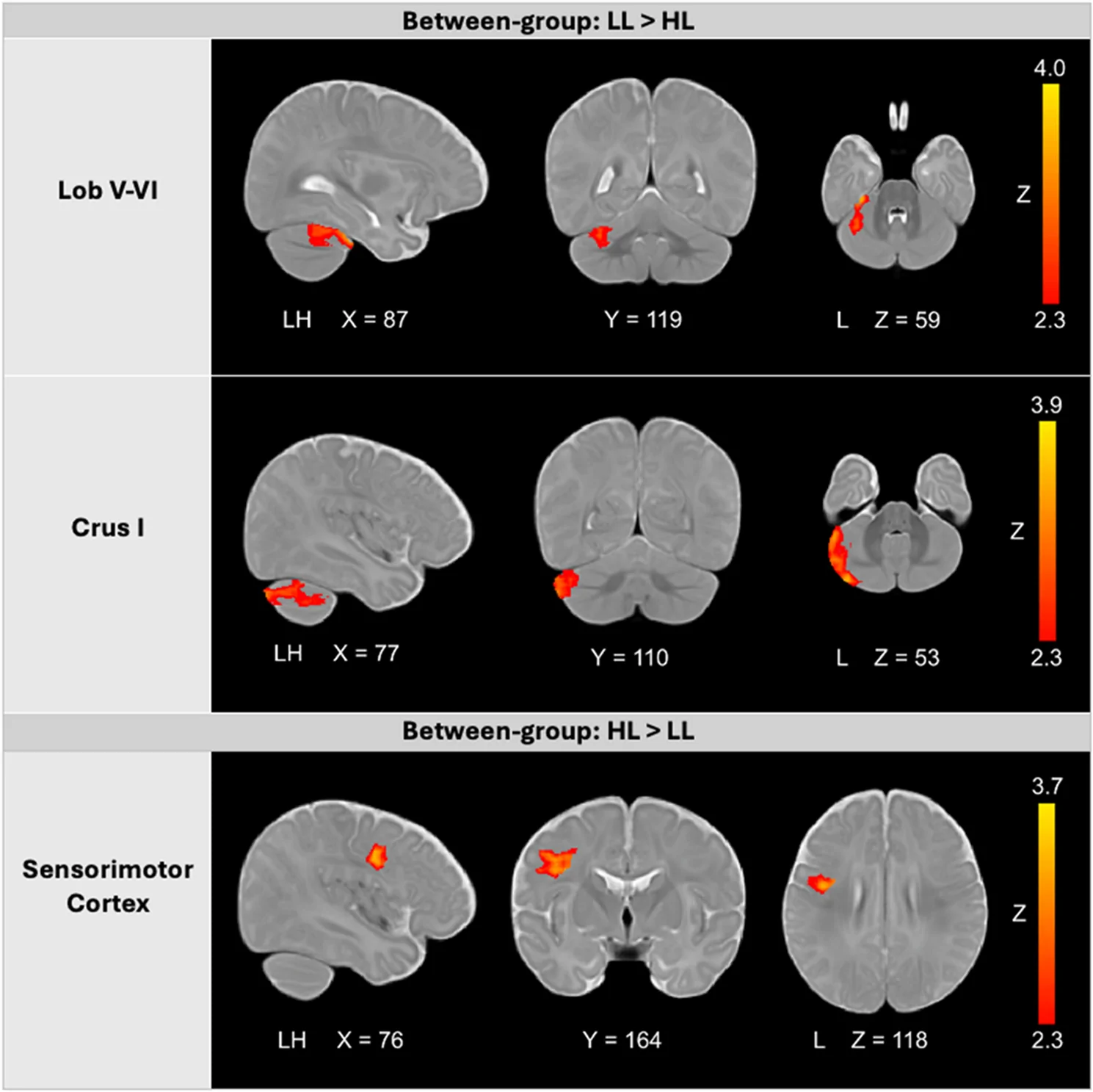
Between-group comparisons of right anterior insula (rAI) connectivity to cerebellar regions lobules V-VI and crus I, as well as sensorimotor cortex. Results were thresholded at Z > 2.3 and used a cluster-defining threshold of *p* < 0.05. X, Y, and Z denote the voxel coordinates corresponding to the sagittal, coronal, and axial planes, respectively

Prior work has shown altered SN connectivity with sensorimotor regions in HL infants [47] and altered cerebellar connectivity in infants with delayed language trajectories, many of whom had a family history of ASD [72]. Therefore, we used an a priori approach and focused our analyses on seven regions of interest: left and right sensorimotor cortex, bilateral cerebellum lobules I-IV, bilateral cerebellum lobules V-VI, bilateral cerebellum lobules VIIIA/B, left and right cerebellum crus I. Between-group comparisons revealed that LL infants showed stronger rAI connectivity with the left cerebellum crus I and left lobules V-VI in comparison to HL infants. This reflects weaker connectivity between the rAI and cerebellar regions involved in sensorimotor processing and higher-order cognition in infants at elevated liability for ASD. In addition, HL infants showed stronger rAI connectivity with the left sensorimotor cortex in comparison to LL infants (Fig. 3; see Table 3 for coordinate table), potentially reflecting altered prioritization of sensory information. This association was also present with the right sensorimotor cortex at lower thresholds (Z > 1.7, cluster-defining threshold *P* < 1; Supplementary Fig. S4).

**Table 3:** Coordinate table for functional connectivity peaks in group comparisons and behavioral regressions

			Peak (voxel coordinates)			
	Cluster location	L/*R*	Max Z	x	y	z
LL > HL	Crus I	L	3.94	-40.7	-31.6	-32.7
LL > HL	Lobule V-VI	L	4.07	-24.7	-19.6	-28.7
HL > LL	Sensorimotor cortex	L	3.72	-33.5	-4.4	25.8
HL, ISOP + at 6 mo	Sensorimotor cortex	R	3.02	18.6	-19.6	53.9
HL, ISOP + at 12 mo	Lobule VIIIA/B	L	2.94	-12.6	-46	-39.1
HL, SOR + at 12 mo	Sensorimotor cortex	L	3.9	-35.1	-22	47.5
HL, SOR + at 12 mo	Crus I	R	3.13	27.5	-46	-27.1
LL, ISOP – at 6 mo	Crus I	R	3.85	28.3	-53.2	-34.3
LL, SOR – at 12 mo	Lobule I-IV	L	3.52	-15.8	-24.4	-22.3
HL > LL, SOR at 12mo	Lobule V-VI	L	4.91	-18.2	-24.4	-23.9
HL, SOR + at 12 mo	Lobule V-VI	L	3.55	-16.6	-40.4	-25.5
LL, SOR – at 12mo	Lobule V-VI	L	4.78	-19	-25.2	-23.9

### Sleep-onset problem associations with rAI connectivity

**Fig. 4:**
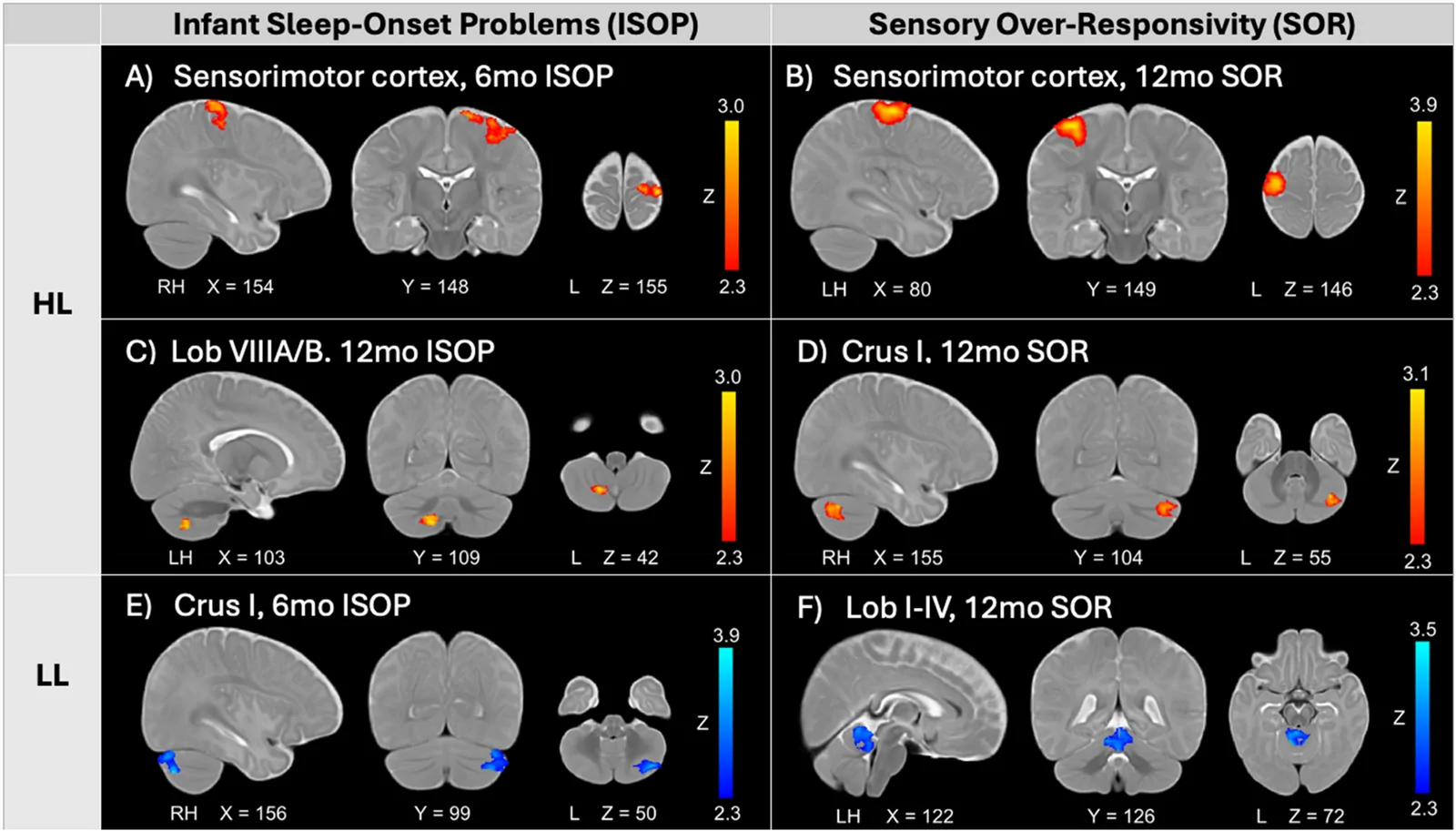
rAI connectivity with a priori regions of interest as a function of Infant Sleep-Onset Problems (ISOP) at 6 and 12 months, and Sensory Over-Responsivity (SOR) at 12 months. Warm color scale indicates positive relationships between connectivity and behavioral scores (i.e., stronger connectivity associated with worse ISOP or SOR). Cool color scale indicates negative relationships between connectivity and behavioral scores (i.e., weaker connectivity associated with worse ISOP or SOR). Contrasts were thresholded at Z > 2.3 and used a cluster-defining threshold of *p* < 0.05. X, Y, and Z denote the voxel coordinates corresponding to the sagittal, coronal, and axial planes, respectively

To determine whether rAI connectivity with our regions of interest was associated with sleep-onset problems, ISOP scores (collected at 6 and 12 months) were entered as regressors of interest in group connectivity analyses. In HL infants, stronger rAI connectivity with right sensorimotor cortex was associated with worse sleep-onset problems at 6 months (Fig. 4A). This association was also present with the left sensorimotor cortex at lower thresholds (Z > 1.7, cluster-defining threshold *P* < 1; Supplementary Fig. S4). Furthermore, in HL infants, there was a positive association between rAI connectivity with left lobules VIIIA/B and ISOP scores at 12 months, such that stronger rAI connectivity to lobules VIIIA/B related to worse sleep-onset problems (Fig. 4C). This association held after controlling for SOR, suggesting that sleep-onset problems at 12 months show a distinct relationship with rAI connectivity to lobules VIIIA/B (Supplementary Table S4). In LL infants, there was a negative association between rAI connectivity with right crus I and ISOP scores at 6 months, such that weaker rAI-crus I connectivity was associated with worse sleep-onset problems (Fig. 4E). These findings indicate that in HL infants, stronger rAI connectivity to sensory processing cortical regions (i.e., right sensorimotor cortex) was associated with worse concurrent sleep-onset problems. Furthermore, stronger rAI connectivity to cerebellar sensory learning regions (i.e., left lobules VIIIA/B) was linked to later sleep-onset problems in HL infants. In contrast, stronger rAI connectivity to a higher-order cerebellar region (i.e., right crus I) was related to fewer concurrent sleep-onset problems in LL infants.

### Sensory over-responsivity associations with rAI connectivity

**Fig. 5:**
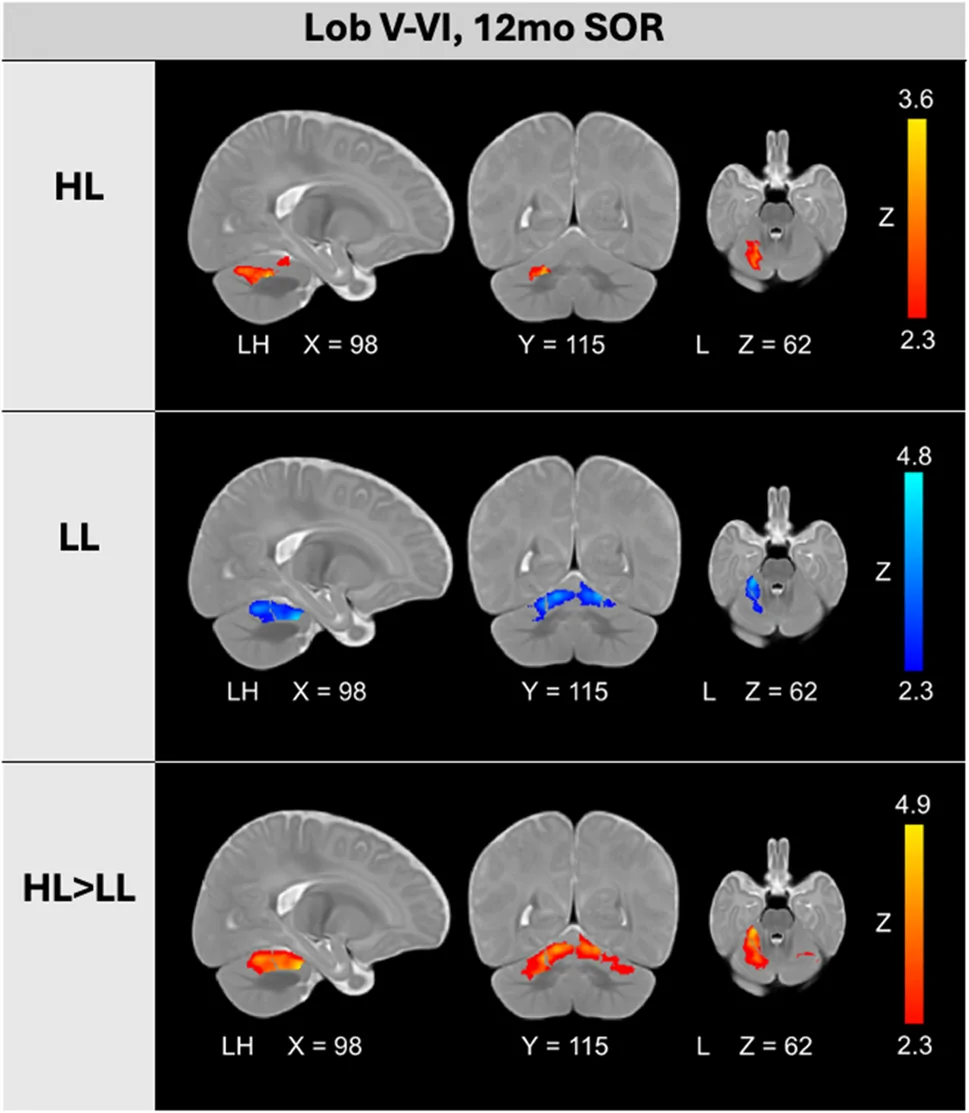
Group interaction of rAI connectivity with cerebellar lobules V-VI and Sensory Over-Responsivity (SOR) at 12 months. Warm color scale indicates positive relationships between connectivity and behavioral scores (i.e., stronger connectivity associated with worse SOR). Cool color scale indicates negative relationships between connectivity and behavioral scores (i.e., weaker connectivity associated with worse SOR). Contrasts were thresholded at Z > 2.3 and used a cluster-defining threshold of *p* < 0.05. X, Y, and Z denote the voxel coordinates corresponding to the sagittal, coronal, and axial planes, respectively

**Fig. 6:**
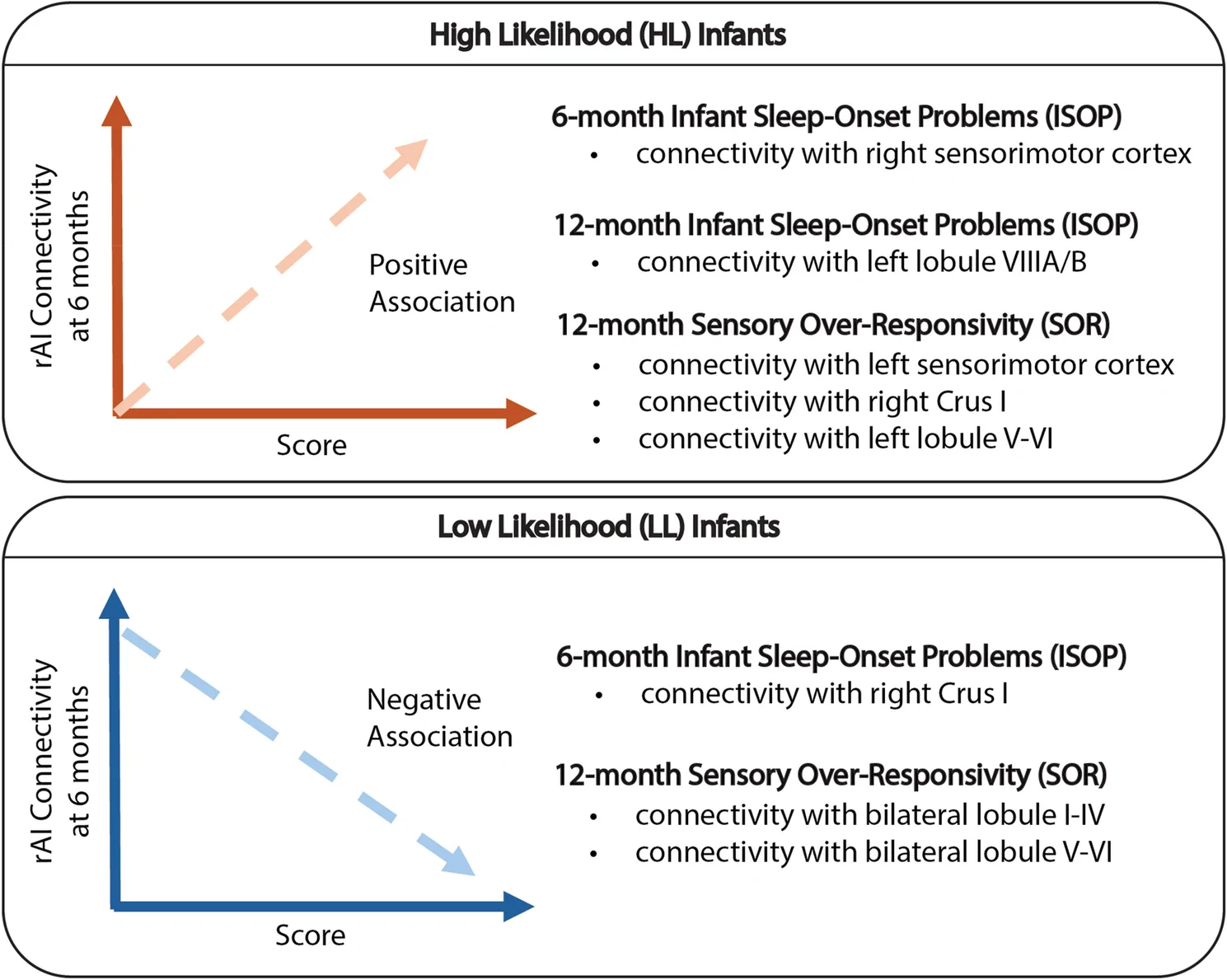
Summary of connectivity findings. High likelihood (HL) infants showed positive associations between right anterior insula (rAI) connectivity with cortical and cerebellar regions and behavioral scores (ISOP or SOR). Low likelihood (LL) infants showed negative associations between rAI connectivity and behavioral scores (ISOP or SOR)

SOR scores (at 12 and 24 months) were used as regressors of interest to examine associations with rAI connectivity. In HL infants, stronger rAI connectivity with left sensorimotor cortex was related to greater SOR severity at 12 months of age (Fig. 4B). This association was also present in the right sensorimotor cortex at lower thresholds (Z > 1.7, cluster-defining threshold *P* < 1; Supplementary Fig. S4). Additionally, stronger rAI connectivity with right crus I was associated with greater SOR severity at 12 months in HL infants (Fig. 4D). In LL infants, weaker rAI connectivity with lobules I-IV was linked to worse SOR severity at 12 months (Fig. 4F). Finally, there was a significant group interaction between rAI connectivity with lobules V-VI and 12-month SOR (Fig. 5). This interaction reflected that HL infants had a positive association between rAI connectivity with lobules V-VI and 12-month SOR, with stronger connectivity relating to worse SOR severity. In contrast, LL infants had a negative association between rAI connectivity with lobules V-VI and 12-month SOR, with weaker connectivity relating to worse SOR severity. When these associations were examined with the broader SEQ hyper-responsivity scores, some clusters were less spatially extensive or present only at lower thresholds (Z > 1.7), which suggests that these relationships are driven specifically by SOR rather than sensory hyper-responsivity more broadly (Supplementary Fig. S5). In addition, these results (except for the positive association between rAI connectivity with lobules V-VI and 12-month SOR in HL infants, Fig. 5) held after controlling for ISOP, indicating that SOR is distinctly associated with rAI connectivity across HL and TL infants (Supplementary Table S4). No associations were observed with 24-month SOR. Overall, stronger rAI connectivity to sensory processing cortices (i.e., left sensorimotor cortex) and cerebellar regions involved in sensory prediction (i.e., lobules V-VI) and higher-order functions (i.e., right crus I) were associated with later SOR in HL infants. In contrast, stronger insular connectivity with some overlapping and distinct regions of the sensory learning cerebellum (i.e., lobules I-IV, V-VI) related to lower SOR at toddlerhood in LL infants. A summary of the connectivity findings can be found in Fig. 6.

## Discussion

In the present study, we investigated functional connectivity of the right anterior insula (rAI) – a hub of the salience network (SN) – with sensorimotor cortices and cerebellum, as a possible mechanism partly underlying sleep-onset problems and sensory over-responsivity (SOR) in 6-month-old infants at high familial likelihood for ASD. Overall, HL and LL infants differed in their rAI connectivity with sensorimotor cortex, cerebellar lobules V-VI, and crus I. We also examined how rAI connectivity patterns related to sleep-onset problems and SOR. LL infants with milder sleep-onset problems and lower SOR demonstrated stronger rAI connectivity to cerebellar sensorimotor regions (i.e., lobules I-IV, V-VI) and crus I. Interestingly, HL infants displayed a different pattern of association whereby infants with worse sleep-onset problems and greater SOR had stronger rAI connectivity with sensorimotor regions (i.e., sensorimotor cortex, cerebellar lobules V-VI, VIIIA/B) and cerebellum crus I.

We first examined associations between sleep-onset patterns, sensory processing, and ASD traits in HL infants. Consistent with our predictions, HL infants displayed a significant positive association between sleep-onset problems and SOR at 12 months, such that worse sleep-onset problems were correlated with more severe SOR (Fig. 2A). This is in line with findings in older ASD youth and adults [27, 91, 111], and previous reports in children as young as toddler and preschool age [23, 26, 28]. Our findings indicate that co-occurrence of sleep problems and sensory challenges manifests earlier in development than previously shown. Furthermore, worse sleep onset problems at 6 months (Fig. 2B) and higher SOR at 12 months (Fig. 2C) both were associated with greater ASD traits at 24–36 months in HL infants. While conclusions on directionality of symptom presentation cannot be made due to the study design, our findings suggest that altered sleep and sensory processing in HL infants might precede ASD symptoms 1-1.5 years later. These results support retrospective reports of early sleep problems that present prior to overt ASD symptoms [2] and converge with previous literature showing a relationship between early sensory challenges and later ASD traits [21, 112–114]. Although HL and LL infants did not differ in overall sleep-onset challenges or SOR, the HL group exhibited greater variability in sleep-onset problems, reflecting increased behavioral heterogeneity. This variability aligns with the diverse outcomes observed in HL infants – while some HL infants will later receive an ASD diagnosis, others may show developmental delays [50, 115], subclinical traits, or typical development. The lack of group differences in SOR might be attributed to challenges that parents may face in reporting a child’s SOR in early life, prior to language acquisition which would allow a child to verbalize discomfort for certain sensory stimuli. Despite comparable mean levels of sleep and sensory challenges, the groups significantly differed on ASD traits, as assessed by the ADOS (Fig. 1A), and broader developmental measures (e.g., motor, language, cognitive processing), as assessed by the Mullen (Fig. 1B). These developmental differences, as well as the unique relationship observed between sleep-onset problems and SOR in HL infants, underscore the need to identify early pathways of brain development that shape divergent behavioral development.

Given the well-established link between sleep disruption and sensory challenges in ASD [23, 26–28, 91, 111], our functional connectivity analyses focused on rAI connectivity with the sensorimotor cortex as a potential shared neural mechanism underlying these symptoms. In comparison to LL controls, HL infants displayed stronger rAI connectivity with left sensorimotor cortex (Fig. 3). Interestingly, stronger rAI connectivity with right sensorimotor cortex at 6 months related to worse concurrent sleep-onset problems (Fig. 4A) and connectivity with left sensorimotor cortex related to greater SOR at 12 months in HL infants (Fig. 4B). At a mechanistic level, this atypical pattern of rAI connectivity could result in increased attribution of salience to sensory stimuli in HL infants. Our findings directly corroborate recent work [47], which has shown that HL infants already exhibit stronger insular connectivity with regions involved in sensorimotor processing at 6 weeks old, and that stronger insular connectivity with primary auditory and sensory processing regions may predict greater SOR. Notably, we significantly expand on this prior work by showing that this pattern of insular hyperconnectivity with sensorimotor cortex persists at 6 months of age and is also associated with sleep-onset problems in HL infants. These findings are also in line with prior reports of stronger SN connectivity with sensorimotor cortex in adult patients with insomnia [83] and in ASD youth with SOR [45, 46]. The consistency across these findings suggests that this pattern of connectivity may linger across the lifespan and perhaps relate to sleep problems more broadly. Furthermore, this shared pattern of atypical SN connectivity may underlie “hyperarousal” theories in both ASD and insomnia, whereby heightened arousal to external or internal stimuli may contribute to both core ASD symptoms [116] and sleep difficulties [117]. Yet, SN hyperconnectivity in older individuals has only been investigated in relation to sleep disruption and sensory challenges independently. To our knowledge, this study is the first to directly implicate connectivity between a key SN hub and the sensorimotor cortex as a common neural mechanism underlying both sleep disturbances and sensory processing atypicalities in early development.

Recent work implicating altered sensorimotor cerebellar connectivity in ASD youth with SOR [70] and atypical crus I connectivity in an infant sample with a family history of ASD [72] prompted us to also explore differences in rAI connectivity with the cerebellum. Overall, in comparison to LL controls, HL infants exhibited weaker rAI connectivity with the sensorimotor cerebellum (i.e., lobules V-VI) and with crus I (Fig. 3). Interestingly, lobule VI and crus I have been shown to be intrinsically connected to the SN in neurotypical adults [118]. However, adults with ASD show reduced connectivity between the rostral prefrontal cortex – another node of the SN – and cerebellar lobule VI and crus I [43]. Our findings show that a similar pattern of connectivity is already present in LL infants and disrupted in HL infants, suggesting that aberrant SN-cerebellar connectivity may present as early as 6 months of age in infants at increased likelihood for ASD. Furthermore, our findings indicate that in LL infants, stronger rAI connectivity with cerebellar areas at 6 months are associated with more favorable sleep and sensory outcomes. Specifically, stronger connectivity between the rAI and right crus I was associated with fewer sleep-onset problems at 6 months in LL infants (Fig. 4E). Additionally, stronger rAI connectivity with the sensorimotor cerebellum (i.e., lobules I-IV and lobules V-VI) was associated with less SOR at 12 months in the LL group (Figs. 4F and 5). Given the cerebellum’s important role in error-based and predictive learning [81], increased functional coupling with the rAI may enable early sensory learning which, in turn, may lead to more developmentally appropriate processing of external stimuli. Interestingly, in HL infants, stronger rAI connectivity with some unique and overlapping cerebellar regions was associated with worse SOR and sleep-onset problems at 12 months. Here, stronger rAI connectivity with lobules VIIIA/B related to worse later sleep-onset problems in HL infants (Fig. 4C). In addition, stronger rAI connectivity with the right crus I (Fig. 4D) and lobules V-VI (Fig. 5) were associated with worse SOR. Cerebellar dysfunction at the structural and functional level has consistently been reported in ASD [65, 70, 119–123], potentially triggering atypicalities in error prediction and learning. Canonically known for its role in motor control, the cerebellum utilizes error signals produced through mismatches between true and predicted outcomes to refine its internal model for future predictions [106, 124]. However, increasing evidence shows that the cerebellum’s role in prediction learning applies to other domains beyond motor control [125–127]. Taken together, these results suggest that in HL infants, insufficient predictive learning may hinder sensory habituation and cause heightened reactions to external stimuli. Thus, strengthening of insular-cerebellar networks may support sensory learning in typical development, whereas it may promote sustained, heightened salience of external stimuli in HL infants. The improper integration of these stimuli into an updated prediction model in HL infants might ultimately contribute to SOR and sleep problems.

Overall, these findings suggest that HL infants show atypicalities in the functional networks involved in the detection and filtering of sensory stimuli, and related predictive learning. Given that proper sensory gating is responsible for facilitating and maintaining sleep [35], improper allocation of attention to irrelevant sensory inputs and perturbed sensory prediction may partly interfere with the ability to fall asleep. Indeed, recent work has implicated functional overconnectivity of sensory cortices with the thalamus – the brain’s sensory relay station – in sensory sensitivities in HL infants [62] and sleep problems in toddlers with ASD [23]. However, the etiology of sleep problems in ASD is likely multifactorial (e.g., circadian rhythm disruption, behavioral dysregulation). Therefore, while we identify the rAI as a key region involved in early sleep problems and SOR, we cannot establish a direct causal relationship between altered rAI functional connectivity and sleep disturbances in early life. We show that HL infants exhibit an altered pattern of rAI connectivity that persists at 6 months of age, which may predispose them to directing undue attention to sensory stimuli and interfere with sensory prediction. Disrupted sleep may further compound these vulnerabilities, as proper sleep is crucial for brain development [128]. Thus, implementing early interventions while the brain is still highly plastic may have dramatic effects on later outcomes [129, 130] and scaffold optimal neurodevelopment.

### Limitations

This study has several limitations. First, given the small number of infants who received an ASD diagnosis (*N* = 7), our analyses were focused on the HL group as a whole. Accordingly, our findings offer new insights into altered neurodevelopment in infants at increased familial likelihood for ASD, as opposed to defining differences based on later diagnostic outcome. Second, we focused our analyses on the rAI, which is only one cortical hub of the SN. While prior work investigating the SN has specifically honed in on the rAI [40, 45, 47, 100], future work examining other SN nodes (e.g. anterior cingulate) may provide a more comprehensive understanding of SN connectivity. Third, while parent-reported questionnaires are generally reliable [131] and are the most common method used to assess sleep patterns and sensory processing in infants and young children, the lack of more objective measures presents another limitation. Future research incorporating objective tools, such as actigraphy for sleep [132] and clinician-administered assessments for sensory processing (e.g., Sensory Assessment for Neurodevelopmental Disorders (SAND); [133]) could provide critical insights into the early development of sleep and sensory challenges. Fourth, while sensory processing atypicalities in ASD encompass SOR, sensory hypo-responsivity and sensory seeking behaviors [134], we focused only on SOR given its previously reported associations with sleep problems [24, 25, 91]. Fifth, additional correction across the multiple ROI analyses were not performed given that the ROIs were chosen a priori based on the prior literature. Sixth, this study did not formally assess the shared contributions of sleep-onset problems and SOR to rAI connectivity. Future work examining their shared effects on functional connectivity may further elucidate the extent to which these behaviors reflect overlapping neural mechanisms. Lastly, as in most infant neuroimaging studies to date, the infants’ sleep stage was not assessed during the MRI scan, primarily due to the considerable challenge of collecting simultaneous EEG and MRI data in this population [135]. However, studies collecting MRI data during natural sleep in infants and toddlers take steps to account for this possible confound (e.g., Linke et al., 2023 [23]), which we have addressed in the supplementary materials.

## Conclusions

In summary, by leveraging functional MRI in infants at high and low likelihood for ASD, these findings advance our understanding of how diverging patterns of early brain functional connectivity patterns may underlie sleep and sensory challenges widely observed in ASD. We found that HL infants exhibited atypical rAI connectivity to sensorimotor cortices and cerebellar regions which was associated with more severe sleep-onset problems and sensory over-responsivity. These sleep and sensory processing challenges in HL infants – which were behaviorally linked to one another and to later ASD traits – may share a common neural substrate as early as 6 months of age. This work expands on prior reports of altered SN connectivity associated with ASD and sleep disruption in older individuals and suggests that over-attribution of attention to sensory stimuli and aberrant prediction learning manifest in early infancy.

## Supplementary Information

Supplementary Material 1.

## Data Availability

The data used in these analyses are available via the National Institutes of Mental Health Data Archive (Collections #19 and #2027). Researchers can submit a Data Access Request to access de-identified human subjects data at https://nda.nih.gov/. The dataset supporting conclusions of this article is available via the National Institutes of Mental Health Data Archive study # 2966.
