# Supplementary material for: Early sleep and sensory over-responsivity relate to insular functional connectivity with sensorimotor cortices and cerebellum in infants at varying likelihood for autism
Source: J Neurodev Disord. 2026 Aug 4;18:62. doi: 10.1186/s11689-026-09721-0 (PMC13726208; doi:10.1186/s11689-026-09721-0)
Supplement: Supplementary file 1 [file 11689_2026_9721_MOESM1_ESM.docx]

**Supplementary Material**

**Sleep Stage Control Analyses**

MRI scans were acquired during natural sleep as part of the Infant Brain Imaging Study (IBIS). Due to technical challenges of collecting simultaneous MRI and EEG, sleep stage during scans cannot be conclusively established. Prior work has shown that the amplitude of the global signal (defined as the standard deviation of the global signal) is related to EEG-defined vigilance states^1-2^, with higher amplitude correlated with lower vigilance (i.e., decreased wakefulness). To rule out potential confounds introduced by sleep stage differences, we compared the global signal amplitude between HL and LL groups using a two-sample t-test and found no significant group differences (t = 1.079, p = 0.283). This suggests that vigilance state did not differ between HL and LL infants, and any group differences in functional connectivity are likely not driven by differences in sleep stage.

**Supplemental References**

1. Wong, C. W., Olafsson, V., Tal, O., & Liu, T. T. (2013). The amplitude of the resting-state fMRI global signal is related to EEG vigilance measures. *NeuroImage*, *83*, 983–990. <https://doi.org/10.1016/j.neuroimage.2013.07.057>
2. Horovitz, S. G., Fukunaga, M., de Zwart, J. A., van Gelderen, P., Fulton, S. C., Balkin, T. J., & Duyn, J. H. (2008). Low frequency BOLD fluctuations during resting wakefulness and light sleep: a simultaneous EEG-fMRI study. *Human brain mapping*, *29*(6), 671–682. https://doi.org/10.1002/hbm.20428

**Supplementary Figure S1. Number of functional runs used for analysis for high likelihood (HL) and low likelihood (LL) infants.** No significant difference in number of functional runs between HL and LL infants (Two sample t-test: t = 0.665, p = 0.508). Errors bars denote 95% confidence interval.

**Supplementary Figure S2.** **ROI masks used for between-group comparisons and behavioral regression analyses.** A) Left sensorimotor cortex (light blue) and right sensorimotor cortex (green). B) Left cerebellum crus I (yellow), right cerebellum crus I (red), cerebellum lobule I-IV (orange), cerebellum lobule V-VI (magenta), and cerebellum lobule VIIIA/B (dark blue).

**Supplementary Figure S3. Spearman rank order correlations with Sensory Experiences Questionnaire (SEQ) hyper-responsivity scale.** A) Correlation between Infant Sleep-Onset Problems (ISOP) and hyper-responsivity at 12 months (p = 0.068). B) Significant correlation between 12-month hyper-responsivity and Autism Diagnostic Observation Schedule (ADOS) severity scores at 24-36 months (p = 0.026).

**Supplementary Figure S4. Right anterior insula (rAI) connectivity with bilateral sensorimotor cortex.** Warm color scale indicates positive relationships between connectivity and behavioral scores (i.e., stronger connectivity associated with worse Infant Sleep-Onset Problems (ISOP) or Sensory Over-Responsivity (SOR)). Contrasts were thresholded at Z > 1.7 and used a cluster-defining threshold of P < 1. X, Y, and Z denote the voxel coordinates corresponding to the sagittal, coronal, and axial planes, respectively.

**Supplementary Figure S5. Right anterior insula (rAI) connectivity with *a priori* regions of interest as a function of the Sensory Experiences Questionnaire (SEQ) hyper-responsivity score at 12 months.** Warm color scale indicates positive relationships between connectivity and behavioral scores (i.e., stronger connectivity associated with worse hyper-responsivity). Cool color scale indicates negative relationships between connectivity and behavioral scores (i.e., weaker connectivity associated with worse hyper-responsivity). Contrasts were thresholded at Z > 1.7 or Z > 2.3 and used a cluster-defining threshold of p < 0.05. X, Y, and Z denote the voxel coordinates corresponding to the sagittal, coronal, and axial planes, respectively.

**Supplementary Table S1.** **Infant Sleep-Onset Problems (ISOP) score**. Five questions from the Infant Behavior Questionnaire (collected at 6 and 12 months) were used to generate the Infant Sleep-Onset Problems (ISOP) score. Questions were scored on a 7-point scale ranging from “never” to “always”. R indicates reverse-scored items. Higher scores indicate worse sleep-onset problems.

| **When going to sleep at night, how often did your baby:** | |
| --- | --- |
| 21. Fall asleep within 10 minutes? | R |
| 22. Have a hard time settling down to sleep? |  |
| 23. Settle down to sleep easily? | R |
| **When your baby awoke at night, how often did s/he:** | |
| 24. Have a hard time going back to sleep? |  |
| 25. Go back to sleep immediately? | R |

**Supplementary Table S2.** **Sensory Over-Responsivity (SOR) score**. Eleven questions from the Sensory Experiences Questionnaire (collected at 12 and 24 months) were used to generate the Sensory Over-Responsivity (SOR) score. Questions were scored on a 5-point scale ranging from “Almost Never” to “Almost Always.” Higher scores indicate greater frequency or intensity of sensory features.

**Experiences with Sound:**

1. Does your child react sensitively or startle easily to unexpected or loud sounds?

5. Does your child notice sounds in the environment (such as planes, trains, faucets dripping, lights buzzing, etc.) before other people do?

6. Does your child show distress (startles, covers ears, etc.) during loud conversations or singing?

**Experiences with Sight:**

8. Is your child disturbed by too much light inside or brightness outside?

11. Does your child avoid looking at your face during social games/play?

**Experiences with Touch:**

14. Does your child dislike cuddling or being held?

15. Does your child show distress during grooming? (For example: cries or fusses during face washing, hair combing, fingernail cutting, or teeth brushing.)

16. Does your child avoid touching certain textures (such as fuzzy or squishy toys) or playing with messy materials (such as sand, lotion)?

17. Does your child react negatively or pull away when touched by a person? (For example: pulls away when head is patted.)

18. Does your child have trouble adjusting to the water temperature during bath time for does he/she dislike being in water?

20. Does your child dislike being tickled?

**Supplementary Table S3. Follow-up regression analyses.** Linear regressions assessing the relationship between Infant Sleep-Onset Problems (ISOP), Sensory Over-Responsivity (SOR), and Autism Diagnostic Observation Scale (ADOS) severity scores while controlling for Mullen Early Learning Composite (ELC) Scores.

|  | **Coefficient** | **SE** | **P-value** |
| --- | --- | --- | --- |
| **ISOP Score – 6 months** | 0.449 | 0.228 | 0.05491 |
| **SOR Score – 12 months** | 0.221 | 0.527 | 0.00012 |

**Supplementary Table S4. Follow-up control analyses.** Coordinate table for functional connectivity peaks (voxel coordinates: x, y, z) in control analyses conducted with Infant Sleep-Onset Problems (ISOP) and Sensory Over-Responsivity (SOR) scores included in the same models.

|  | **Cluster Location** | **L/R** | **Max Z** | **x** | **y** | **z** |
| --- | --- | --- | --- | --- | --- | --- |
| **12-month ISOP controlling for 12-month SOR** | | | | | | |
| HL, ISOP+ | Lobule VIIIA/B | L | 2.99 | -11 | -46.8 | -39.1 |
| **12-month SOR controlling for 6-month & 12-month ISOP** | | | | | | |
| HL, SOR+ | Sensorimotor cortex | L | 3.26 | -27.9 | -6.8 | 51.5 |
| HL, SOR+ | Crus I | R | 3.39 | 34.7 | -50 | -33.5 |
| LL, SOR- | Lobule I-IV | L | 3.08 | -6.21 | -38.8 | -14.3 |
| LL, SOR- | Lobule V-VI | R | 3.31 | 11.4 | -41.2 | -14.3 |
|  | Lobule V-VI | L | 4.56 | -19 | -24.4 | -23.9 |
| HL>LL, SOR | Lobule V-VI | L | 4.5 | -18.2 | -24.4 | -23.9 |
